# Smoking and health-related quality of life in English general population: implications for economic evaluations

**DOI:** 10.1186/1471-2458-12-203

**Published:** 2012-03-19

**Authors:** Matthias Vogl, Christina M Wenig, Reiner Leidl, Subhash Pokhrel

**Affiliations:** 1 Helmholtz Zentrum München, German Research Centre for Environmental Health, Member of the German Centre for Lung Research, Institute of Health Economics and Health Care Management, Ingolstädter Landstrasse 1, 85764 Neuherberg, Germany; 2 Ludwig-Maximilians-Universität München, Institute of Health Economics and Health Care Management & Munich Centre of Health Sciences, Ludwigstrasse 28, 80539 Munich, Germany; 3 Health Economics Research Group, Brunel University, West London, UK

**Keywords:** Health-related quality of life (HRQoL), EQ-5D, EuroQol, Socio-economic determinant, Economic evaluation

## Abstract

**Background:**

Little is known as to how health-related quality of life (HRQoL) when measured by generic instruments such as EQ-5D differ across smokers, ex-smokers and never-smokers in the general population; whether the overall pattern of this difference remain consistent in each domain of HRQoL; and what implications this variation, if any, would have for economic evaluations of tobacco control interventions.

**Methods:**

Using the 2006 round of Health Survey for England data (n = 13,241), this paper aims to examine the impact of smoking status on health-related quality of life in English population. Depending upon the nature of the EQ-5D data (i.e. tariff or domains), linear or logistic regression models were fitted to control for biology, clinical conditions, socio-economic background and lifestyle factors that an individual may have regardless of their smoking status. Age- and gender-specific predicted values according to smoking status are offered as the potential 'utility' values to be used in future economic evaluation models.

**Results:**

The observed difference of 0.1100 in EQ-5D scores between never-smokers (0.8839) and heavy-smokers (0.7739) reduced to 0.0516 after adjusting for biological, clinical, lifestyle and socioeconomic conditions. Heavy-smokers, when compared with never-smokers, were significantly more likely to report some/severe problems in all five domains - mobility (67%), self-care (70%), usual activity (42%), pain/discomfort (46%) and anxiety/depression (86%) -. 'Utility' values by age and gender for each category of smoking are provided to be used in the future economic evaluations.

**Conclusion:**

Smoking is significantly and negatively associated with health-related quality of life in English general population and the magnitude of this association is determined by the number of cigarettes smoked. The varying degree of this association, captured through instruments such as EQ-5D, may need to be fed into the design of future economic evaluations where the intervention being evaluated affects (e.g. tobacco control) or is affected (e.g. treatment for lung cancer) by individual's (or patients') smoking status.

## Background

Smoking is an important health and economic problem. Every year, tobacco related diseases claim the lives of nearly six million people globally and the costs to society of smoking are enormous [1]. Taking a very narrow perspective of the British National Health Service (NHS) alone, the treatment costs of diseases that were attributable to smoking in the year 2005/6 was £5.2 billion which is equivalent to 5.5% of the total NHS budget for that year [2]. This figure increases several folds if we add other indirect costs to society, e.g. lost productivity, informal care and costs due to passive smoking. For example, total costs attributable to smoking in Germany in 2003 were €21.0 billion of which direct costs amounted to only €7.5 billion (36% of total) [3]. If the same proportion were true in the UK context, the total costs attributable to smoking in the UK would be more than £14.6 billion (2005/6). The adverse effects of smoking, particularly in terms of causing many preventable diseases and premature deaths, have widely been discussed, and more recently, smoking has been a burning issue in the debate around health inequalities as it has been shown to have accounted for a significant proportion of health inequalities [4]. Therefore, importance is being given to several public health strategies that aim to increase the quitting rates of current smokers as well as regulations aiming at the prevention of health burden due to passive smoking. Worldwide public health initiatives aim to prevent and reduce both the prevalence and consequences of smoking [5]. In England alone, for example, the average prevalence of smoking for those aged 16 years and above has fallen from 27% [6] to 21% in 2008 [7] since the landmark public health strategy on tobacco control, *Smoking Kills *came into being in 1998 [8]. The National Institute for Health and Clinical Excellence (NICE) has published a public health guidance on smoking cessation services [9]. Despite these efforts, due to high rates of relapse [10] and incidence of new smokers, smoking remains as a major public health challenge in the years to come.

An important aspect of smoking is its association with health-related quality of life (HRQoL). Smoking not only kills, it affects individuals' (current, ex- and passive smokers') quality of life too. In the UK, for example, it is estimated that 19% of all deaths in 2002 were due to smoking (27% in men and 11% in women) but it was also found to be directly responsible for 12% of disability adjusted life years lost in that year [2]. There are only a few studies that explore the relationship between smoking and health-related quality of life in the general population. Additional file 1 summarizes these studies. It is important to note that these studies differ widely in the way they have measured both HRQoL and smoking status. Nevertheless, the message appears to be consistent across all studies, i.e. smokers are likely to have worse health-related quality of life.

There is a dearth of relevant data that could be used to inform economic evaluations of interventions that affect (e.g. tobacco control) or are affected by population smoking status (e.g. treatment of lung cancer). Examining the extent to which smoking is associated with health-related quality of life may help the future studies that look at the cost-effectiveness of interventions. For example, limited information exists on the utility loss due to smoking [e.g. [11]] but it is not clear whether adjusting for other biological, clinical, lifestyle and socioeconomic conditions would lead to the same level of utility loss, particularly in the context that a clear socioeconomic gradient exists within smoking population [12]. The utility loss is an important input to any cost-utility analysis. Further, the information on how utility loss from smoking varies by age, gender and socio-economic status could also be used as inputs to subgroup analysis in the economic evaluation of, say smoking cessation interventions. It is interesting to note from Additional file 1 that of those limited number of studies that explored the association between HRQoL and smoking status, only three studies used EQ-5D as a measure of health-related quality of life. Given that EQ-5D is being widely used in economic analyses and is the recommended instrument to measure HRQoL in economic evaluations by the National Institute for Health and Clinical Excellence (NICE) in the UK [13], this clearly calls for more research in this area.

In this paper, we estimate the net association of smoking status on health-related quality of life, as measured by EQ-5D, in the English general population. EQ-5D is an instrument that captures five dimensions - mobility, self-care, usual activities, pain/discomfort, anxiety/depression - each of which can take one of three responses - no problems; some or moderate problems and extreme problems [14]. The estimated effects are then used to predict values that can be used as 'utilities' attached to each smoking status in order to inform the future economic evaluations. A secondary aim is to find out which dimensions of HRQoL are affected by smoking, and if so, to which degree they are affected.

## Methods

We used the 2006 round of Health Survey for England (HSE), available for download from the UK Data Archive (http://www.data-archive.ac.uk). The HSE is a series of surveys intended to monitor trends in the nation's health [15]. It is a representative national survey of the population living in private households in England in which all adults aged 16 years or older at each household were selected for the interview. Seasonal differences were taken into account by conducting interviews throughout the year. In 2006, all adults were asked questions on cardiovascular diseases, general health, smoking, alcohol consumption and physical activities. The survey included adults (16 and above) and children in the general population. This study focuses on valid adult (16 years and above) observations only (n = 13,241). A multi-stage stratified random sampling was followed to recruit the participants whose data was collected by using a mix of methods (face-to-face interviews, self-completion questionnaires, and clinical measurements). A detailed description of the survey is provided elsewhere [16].

### HRQoL and smoking status variables

The HSE collected data on health status by using the EQ-5D instrument which describes health in five dimensions (mobility, self-care, usual activity, pain/discomfort, anxiety/depression) and a single value (tariff) for each health state can be obtained using a standardized formula [15]. This type of measure is of growing relevance in assessing public health issues [17,18] and it is one of the most qualified and common instruments used in population based studies on HRQoL [19]. The EQ-5D is a widely used instrument in measuring the loss in HRQoL by the presence of diseases caused by smoking such as COPD [20], cancer [21] and heart diseases [22,23].

The HSE also collected individuals' response to questions related to smoking status. This allowed us to assign the respondents in our sample to one of the following six smoking groups: never-smokers (one who has never smoked), ex- occasional smoker (one who has only smoked once or twice), ex-regular smoker (one who used to smoke sometimes but never smoke a cigarette now), light smoker (one who smokes under 10 cigarettes a day), moderate smoker (one who smokes between 10-19 cigarettes a day), and heavy smokers (one who smokes 20 or more cigarettes a day). The advantage of this approach in smoking status compared to two (non vs. current) or three levels (never-, ex- and current), as widely used in the literature, is its ability to generate more granularity in the scrutiny of how HRQoL varies by smoking status. Results using a more general classification of the smoking status variable are available from the authors.

### Modelling EQ-5D tariff data

As EQ-5D measure has two different types of data (i.e. tariff or single value/mean score and dimensions), it was important to acknowledge the nature of these data and apply appropriate statistical methods. Both types of data are problematic and therefore special considerations were given as described below.

The tariff data suffers from a skewed and censored distribution as a large proportion of the individuals have a tariff equal to 1 (the highest possible value), has a gap between the value of 0.883 and 1, and in some samples may be multi-modal [24]. This indicates that alternative models dealing with that special nature of data need to be taken into account. Empirical evidence, largely coming from the mapping exercise based on specific patient-group samples, suggest that Tobit and censored least absolute deviation (CLAD) estimators may be more appropriate than ordinary least squares [25-27], accounting for the fact that full health in terms of HRQoL might actually exceed 1, but we do not observe any such values in real life [28,29]. However, none of these estimates can handle all of the above properties of tariff data. For this reason, several studies based on population-based data have also reported estimates from ordinary least squares (OLS) or its variants [30,31].

Other types of models such as interval regression, quantile regression and ordinal regression [32], are potential candidates but in order to answer the research questions such as the ones in this paper, they are not very helpful. These methods require that assumptions about cut-off points in the tariff data be made, which although provides some interesting statistical properties to fit the models, are unrealistic and often result in difficulty in interpreting the findings. One of the arguments in favour of using OLS in mapping exercise that uses specific patient-group data is that the upward censoring of the EQ-5D tariff at 1, as argued in the Tobit or CLAD model, is hardly observed in any real data [33]. However, this may not be applicable in large population-based data such as ours. In our sample, about 58% individuals had the EQ-5D tariff value of 1, suggesting the OLS approach might be inappropriate and we needed to explore alternative approaches. Two-part models [27] dealing with the censored part separately are difficult to interpret in relation to economic evaluations. This may also lead to further problems not broadly addressed in the literature yet (e.g. the choice of appropriate models in two-part construct). Therefore, the focus tends to shift towards Tobit and CLAD models which treat the distribution of EQ-5D tariff data as censored at 1, implying the possibility of predicting values greater than 1 [34] and modelling a 'latent' HRQoL [35]. Recent models based on experience-based and decision-utility approaches [36] or beta regression approaches [37] are yet to be picked up by the modelling community.

Acknowledging that there has not been any statistical method which can handle all the above properties of tariff data, we tested three different models: an OLS (as a benchmark), a Tobit (to allow for upward censoring at 1 [29]) and a CLAD (to allow for upward censoring and heteroscedasticity [35]) to establish the association between smoking status and the EQ-5D tariff controlling for other covariates. The choice of covariates was guided by previous research in this area (see Additional file 2). The covariates considered were: (a) biology- age, sex, BMI, presence of cardio-vascular disease, number of limiting conditions; (b) lifestyle - frequency of drinking, participation in physical activity (note smoking is a research variable and described above); (c) socioeconomics - ethnicity, marital status, education, economic status, household equivalised income, household size; and (d) social capital - the extent to which the individual enjoys living in their community (intuitively considered as a simple proxy for social capital). The regression models were subject to standard diagnostic tests, adjustment of clustering within postcode areas and application of sampling weights.

Modelling EQ-5D tariff in the general population (as opposed to patient-group data) has been a challenge and the discussions around how to model it has been controversial in the literature [34,38]. Our own experience mirrored this problem. None of the above models 'performed', as they failed to pass all the diagnostic tests carried out post-estimation. Particular problems included normality assumptions in OLS (e.g. error terms were not normally distributed), off-boundary predicted values in Tobit (i.e. the mean predicted value was greater than 1 compared to mean observed value of 0.8575), and mixed and 'contrary-to-expectation' signs in CLAD (i.e. the coefficient corresponding to heavy-smokers was positive). Therefore, we did not carry out comparative analysis between the models; rather we focussed on three aspects of each model - (a) the overall F-value and significance of each covariate in the models (all models passed this criteria); (b) the signs are as expected a priori, e.g. HRQoL decreases with age, increases with income and decreases with number of longstanding illnesses (OLS performed the best in this criterion); and (c) the ability of the model to predict 'adjusted' EQ-5D tariff. On this basis and as the paper was intended to inform economic evaluations, OLS was found to be the most useful model and therefore all subsequent analysis on the tariff is based on OLS.

### Modelling EQ-5D dimensions data

Due to the nature of dimensions data (ordinal), different statistical approach was needed. In our sample, there was small number of observations in the "severe problems" groups of the EQ-5D dimensions, mainly because this is a population-based data and we would expect a small number of people having severe problems. This did not allow us to apply ordinal regression (or multinomial logistic regression if one rejects the notion of ordinality in such data) as these models did not converge or if they did, did not pass the tests for underlying assumptions of these models, e.g. tests for parallel lines. Therefore, in order to be meaningful and consistent with the literature [30,39,40] the "some problems" and the "severe problems" group in each dimension were combined in order to use binary logistic regression models with two categories: "no problems" and "some/severe problems". In order to be consistent, the set of co-variates was the same as that in the models of tariff data. The *Hosmer-Lemeshow *test was used to determine that the model fit the data [41]. For interpretation purposes, the odds ratios yielded by these models are treated as relative risks, provided the event (having problems in any dimension) is rare (< 10% of the total sample size) and estimated odds ratios are close to 1 [42].

### Assessing implications of missing data

Another complication in statistical analyses was the missing data. There were some missing values on EQ-5D measures (the dependent variable), smoking status (the research variable) and others (the confounders). We did a number of assessments to judge the implications this would have for the findings. A total of 315 individuals out of 13,241 (2.38%) had missing EQ-5D data. In order to establish the extent to which omitting these 315 observations from our analysis would make any difference, we modelled the probability of having a missing EQ-5D tariff on all covariates we are interested in. The underlying assumption was that if this probability had not been determined significantly by any of the covariates we were interested in our main analysis, excluding these missing observations would have been reasonable. A logistic regression was applied, and after all diagnostic tests, it was determined that we could afford losing 315 observations from our main analysis. Thus, imputing the values for missing data and including those in the main analysis was not the preferred option as this would induce another bias in the analysis. The model outputs on the missing data analysis are not reported here but available from the authors upon request.

We also observed a large number of missing values in a few covariates (e.g. equivalised income -18%; BMI - 12% missing) and this needed a close scrutiny. We chose not to impute missing data because it was difficult to assess the nature of missingness and any attempt to impute values was more likely to widen confidence interval around the estimates. Instead, we opted to run the analysis on all 12,926 observations with complete EQ-5D but retained an additional category on each covariate (where a large number of data was missing) to allow for the observations with missing data. For example, we had 6 categories of income variable: 5 quintiles of income plus a category to indicate missing data. All analyses were performed using STATA 11.1 software.

## Results

The descriptive statistics of our sample is provided in Additional file 3. In particular, the mean EQ-5D tariff was 0.8575 (sd = 0.2316) and the percentage of individuals who reported some or severe problems in EQ-5D domains varied: mobility (18.1%); selfcare (5.4%); usual activity (16.4%); pain and discomfort (32.5%); and anxiety and depression (18.2%). Less than half of the sample (47%) never smoked cigaretts, 26% were ex-regular smokers, and about 22% were current smokers. About 6% currently smoked 20 or more cigaretts a day (classified as heavy smokers). The mean age of the sample was 48.9 years (sd = 18.3), 55.6% were females and just more than half (55.7%) were in employment.

Table 1 presents the observed mean value for EQ-5D tariff and observed frequency of reporting some or severe problems in the five EQ-5D domains by smoking status. It is important to note that the observed value for 'heavy smokers' (0.7739) is about 12 percentage points lower than that of the never-smokers (0.8839). As expected, the difference in tariff values between never-smokers, ex-occasional smokers and light-smoker is not large (0.8839 to 0.8724). A difference in those values indicates the magnitude of decline in HRQoL in smokers compared with never-smokers. Domainwise, heavy smokers are much more likely to report a problem in all EQ-5D domains compared to never-smokers. This ranged from 49% in pain/discomfort to 149% in self-care (p-value < .001). On the face of the observed statistics, there is thus enough indication that smoking is significantly associated with HRQoL, as measured by EQ-5D.

**Table 1:** Observed values of HRQoL by smoking status (N = 13,241) in English general population

Smoking status	% of N	Mean*	SD*	Range*	M**	SC**	UA**	PD**	AD**
Never smoker	46.9	.8839	.2069	-.484,1	15.04	4.19	13.66	28.20	15.26

Ex-occasional smoker	5.4	.8825	.1891	-.016,1	16.86	4.13	15.48	30.65	16.05

Ex-regular smoker	26.0	.8225	.2512	-.594,1	25.49	7.46	21.62	41.76	18.99

Light smoker	7.2	.8724	.2187	-.184,1	13.38	4.90	11.73	29.56	20.15

Moderate smoker	8.6	.8474	.2432	-.239,1	17.17	5.45	16.65	32.21	23.91

Heavy smoker	5.9	.7739	.3088	-.594,1	26.70	10.42	25.06	41.90	31.92

* *Observed EQ-5D tariff*

** *Observed frequency (%) of "some" or "severe" problems in EQ-5D dimensions*

M = Mobility; SC = Self-care; UA = Usual Activity; PD = Pain/discomfort;

AD = Anxiety/depression)

Based on Health Survey for England 2006

The bivariate analysis however would not tell us whether this observed variation is the 'net effect' of smoking. Table 2 provides partial results from a multivariate analyses that controlled for the effects of a number of variables that are expected to confound the observed values (biology, lifestye, socioeconomics, and social capital). As explained in the methods section, the tariff eqation was estimated using OLS, Tobit and CLAD estimators but all further analyses were based on the OLS and therefore we present OLS results only. Full OLS results are provided in Additional file 4 and other results are available from the authors upon request. The results indicate that there is an apparent smoking gradient in HRQoL as measured by EQ-5D, *ceteris paribus*. After allowing for other covariates, the greatest effect is observed with heavy-smokers compared to never-smokers (beta of -0.0516, p-value < .005) and all forms of smoking have negative gradients. While the unadjusted (observed) utility loss between never-smokers and heavy-smokers was -0.1100, the 'net' loss due to smoking after controlling for all potential covariates was much smaller (-0.0516) but still significant (Table 2).

**Table 2:** The strength of association between HRQoL and smoking status (N = 12,887)

Smoking status	Mean score*	M**	SC**	UA**	PD**	AD**
Never smoker (Ref)						

Ex-occasional smoker	-.002	0.99	0.88	1.12	1.07	1.11

Ex-regular smoker	**-.017**	**1.18**	1.11	1.11	**1.28**	**1.16**

Light smoker	**-.021**	1.13	1.25	0.95	**1.34**	**1.43**

Moderate smoker	**-.033**	**1.55**	**1.45**	1.37	**1.36**	**1.49**

Heavy smoker	**-.052**	**1.67**	**1.70**	**1.42**	**1.46**	**1.86**

Correlation (predicted, observed)	**0.61**	**0.63**	**0.51**	**0.59**	**0.53**	**0.34**

R-squared (OLS)/pseudo R-squared (logistic regressions)	0.37	0.39	0.38	0.34	0.24	0.11

* *Coefficient (OLS, EQ-5D tariff)*

** *Odds ratio (Logistic regressions, EQ-5D dimensions)*

OLS = Ordinary Least Squares; M = Mobility; SC = Self-care; UA = Usual Activity;

PD = Pain/discomfort; AD = Anxiety/depression. A boldface indicates a significant p-value at < .05

As tariff is a summary measure based on the response to the five domains, we also modelled using logistic regression techniques the probabaility with which a paricular smoking status would predict some or severe problems in each of the five domains. The last five columns in Table 2 summarise the findings. Being a heavy-smoker was associated with a 67% more likelihoold in reporting some/severe problems in mobility *ceteris paribus*; 70% in self-care; 42% in usual activity; 46% in pain/discomfort; and 86% in anxiety/depression - all values significant at p < .005. Former smoking, in particular if one smoked regularly in the past, was associated with some/severe problems in mobility (OR = 1.18, p < .005), pain/discomfort (OR = 1.28, p < .005) and anxiety/depression (OR = 1.16, p < .005) but not with self-care and usual activity.

The data on 'utility' losses because of smoking can be very useful in cost-effectiveness modelling where the intervention being evaluated affects (e.g. tobacco control) or is affected (e.g. treatment for lung cancer) by individuals' (or patients') smoking status. Table 3 presents the model predicted 'utility' values by age-group and gender, disaggregated into all categories of smoking. As these values are 'adjusted' for any other potential factors including clinical conditions, lifestyles, biology and socio-economics, the changes in the utility values from one smoking status to the other (e.g. from never-smoker to heavy-smoker) can be regarded as the net change in 'utility' due to smoking.

**Table 3:** Changes in adjusted 'utility' values (by age and gender) as the result of smoking profile*

Smoking category	Gender		16-24	25-34	35-54	45-54	55-64	65-74	75-100	16-24	25-34	35-54	45-54	55-64	65-74	75-100
Never smokers	Men	Mean	0.9522	0.9487	0.9244	0.8775	0.8163	0.7911	0.7441	0.9665	0.9688	0.9483	0.9022	0.8455	0.8212	0.7744
		SE	0.0036	0.0028	0.0025	0.0031	0.0043	0.0051	0.0052	0.0036	0.0028	0.0024	0.0031	0.0043	0.0050	0.0052
	Women	Mean	0.9258	0.9146	0.9038	0.8646	0.7982	0.7852	0.7102	0.9433	0.9349	0.9257	0.8907	0.8293	0.8154	0.7411
		SE	0.0036	0.0027	0.0025	0.0029	0.0044	0.0048	0.0060	0.0035	0.0027	0.0025	0.0029	0.0044	0.0048	0.0060
Ex-occasional smokers	Men	Mean	0.9483	0.9431	0.9176	0.8704	0.8101	0.7868	0.7412	0.9627	0.9632	0.9414	0.8951	0.8393	0.8169	0.7715
		SE	0.0073	0.0067	0.0065	0.0067	0.0074	0.0079	0.0080	0.0073	0.0068	0.0066	0.0069	0.0075	0.0080	0.0081
	Women	Mean	0.9225	0.9106	0.8991	0.8593	0.7930	0.7814	0.7079	0.9401	0.9309	0.9210	0.8854	0.8241	0.8116	0.7387
		SE	0.0071	0.0066	0.0064	0.0065	0.0072	0.0075	0.0084	0.0071	0.0067	0.0065	0.0066	0.0074	0.0076	0.0084
Ex-regular smokers	Men	Mean	0.9342	0.9306	0.9058	0.8596	0.8020	0.7802	0.7358	0.9485	0.9507	0.9296	0.8843	0.8312	0.8103	0.7661
		SE	0.0054	0.0047	0.0041	0.0042	0.0050	0.0059	0.0059	0.0053	0.0047	0.0041	0.0042	0.0050	0.0058	0.0058
	Women	Mean	0.9084	0.8988	0.8872	0.8479	0.7827	0.7709	0.6987	0.9260	0.9191	0.9091	0.8740	0.8138	0.8012	0.7296
		SE	0.0053	0.0045	0.0041	0.0041	0.0051	0.0057	0.0067	0.0052	0.0044	0.0041	0.0041	0.0051	0.0057	0.0066
Light smokers	Men	Mean	0.9321	0.9266	0.9002	0.8526	0.7917	0.7684	0.7229	0.9464	0.9467	0.9241	0.8773	0.8209	0.7985	0.7532
		SE	0.0060	0.0057	0.0059	0.0063	0.0071	0.0077	0.0079	0.0060	0.0058	0.0059	0.0063	0.0072	0.0076	0.0079
	Women	Mean	0.9063	0.8938	0.8814	0.8418	0.7748	0.7631	0.6896	0.9238	0.9141	0.9033	0.8679	0.8059	0.7934	0.7205
		SE	0.0060	0.0059	0.0059	0.0062	0.0071	0.0073	0.0082	0.0060	0.0059	0.0060	0.0062	0.0071	0.0073	0.0082
Moderate smokers	Men	Mean	0.9211	0.9166	0.8899	0.8422	0.7815	0.7575	0.7112	0.9354	0.9367	0.9137	0.8669	0.8107	0.7875	0.7415
		SE	0.0065	0.0062	0.0060	0.0063	0.0070	0.0079	0.0082	0.0065	0.0062	0.0061	0.0063	0.0070	0.0079	0.0082
	Women	Mean	0.8952	0.8835	**0.8716**	0.8317	0.7648	0.7520	0.6778	0.9128	0.9038	**0.8935**	0.8578	0.7959	0.7822	0.7087
		SE	0.0065	0.0061	**0.0060**	0.0062	0.0070	0.0076	0.0087	0.0065	0.0061	**0.0060**	0.0062	0.0070	0.0076	0.0087
Heavy smokers	Men	Mean	0.9006	0.8971	0.8728	0.8259	0.7647	0.7395	0.6925	0.9149	0.9172	0.8967	0.8506	0.7939	0.7696	0.7228
		SE	0.0093	0.0091	0.0089	0.0090	0.0095	0.0103	0.0104	0.0093	0.0091	0.0089	0.0090	0.0095	0.0103	0.0104
	Women	Mean	0.8742	0.8630	0.8522	0.8130	0.7466	0.7336	0.6586	0.8917	0.8833	0.8741	0.8391	0.7776	0.7638	0.6895
		SE	0.0097	0.0095	0.0093	0.0093	0.0097	0.0104	0.0110	0.0097	0.0095	0.0093	0.0093	0.0098	0.0103	0.0110

*As predicted by the OLS model in the population with no limiting condition

To help interpret the data shown in these tables: the mean EQ-5D tariff for a typical female from the "general population" falling under 35-54 age band who is a moderate smoker is 0.8716 with a standard deviation of 0.006 (first part of Table 3). A typical person in the general population is expected to have 0.74 longstanding illnesses (at least in our sample) and therefore these data are not applicable in cost-effectiveness modelling for a cohort of individuals who have no health condition. The second part of Table 3 provides required data. The same person as above if happens to have no longstanding illness, the utility value will be 0.8935 with a standard deviation of 0.006. The utility estimates reported in Table 3 can be used to support economic evaluation of tobacco related interventions/policies. The estimated utility values not only allow calculating QALYs, but also support modelling their uncertainty via probabilistic sensitivity analysis [43,44]. This can be done by assuming beta-distributed utility values. The parameters of the beta-distribution can be calculated based on the expected value and the standard error reported in Table 3[43].

## Discussion

This is the first study that produces a large number of data relating to health-related quality of life (more precisely, the 'utility' values) by smoking status (i.e. extent of smoking) in English general population. Paucity of this kind of data has left economic evaluation researchers very limited choice in modelling the cost-effectiveness of interventions which affect the recipients' smoking status (e.g. tobacco control) or are affected by it (e.g. treatment of lung cancer). There are very few studies that provide some estimates of utility values by smoking status [11,33,45,46]. Our study differs from this in several ways - our sample size is much larger, we are able to provide granularity in estimates, and the estimates reflect the 'net' effect of smoking controlling for other important covariates including socioeconomics which has been considered by some authors as having more impact on HRQoL than smoking status itself [11]. Both the values themselves and the methods with which such values are estimated in this paper are deemed more robust.

Before we discuss the implications of the estimated 'utility' values for economic evaluations, it is important to examine some methodological issues. First, the 'utility' values are based on EQ-5D mean tariff which is not the 'valuation' of individual's health state *per se *but a reflection of it derived from a standard formula (extrapolated from the original UK valuation exercise) applied to the EQ-5D descriptive system [18]. This is a generic problem of EQ-5D [47]. As long as EQ-5D descriptive system remains one of the recommended tools for use in economic evaluations [13], this issue is of less relevance in the context of this paper.

The second issue relates to how best one could model EQ-5D tariff. We used three different estimators and found that despite the difficulty in translating the unique features of general population tariff data under OLS assumptions, OLS predictions were not only consistent with the observed values but were also more useful than those from Tobit and CLAD in terms of measuring 'utility loss' across different smoking profiles. These predictions could be valuable inputs to estimate QALYs in economic evaluation of different interventions, including tobacco control policies. In addition, the literature suggests that Tobit and CLAD estimations, although may perform well in specific patient-group data, do differ from the OLS in general population and are biased [34,35]. Note however that many earlier studies, mostly based on patient group data as opposed to population data as in our case, have resorted to OLS or its variants [25,30] and therefore it is not unreasonable to present predictions based on OLS estimator. Although there have been some very recent efforts to look at alternative ways in which health utility data could be modelled [24,36,37,48] which is yet to be scrutinized closely by modelling community, we emphasize that future research continues to propose and debate estimation strategies that would take into account all the unique features of EQ-5D tariff data.

The third issue is the nature of the utility values. Our results are based on the general population as opposed to patient group and therefore the utility values are that of the general population, and not that of the specific patient population. This may have implications for economic modelling based on a cohort with specific disease conditions. However, it is important to note that the methods with which these values are derived reflect the net losses in utilities due to the degree of smoking. That is, these losses have already been adjusted for one's biology, clinical conditions, lifestyles and socioeconomics (see Additional file 4 for the impact of limiting conditions). It is then up to the economic modeller to decide appropriate states in the Markov model that allows the use of such data in a specific patient group.

There are a number of implications of our findings. The most resounding conclusion that can be drawn from this study is that smoking is significantly associated with HRQoL in English general population. This is consistent with studies reported from similar high-income, industrialized countries such as Spain [49], Finland [50], Australia [51], the Netherlands [52], USA [46,53], Denmark [54], and also with earlier UK studies [11,45,55]. In quantitative terms, moving from never-smoking to heavy-smoking profile leads to a utility loss of 0.0516. Likewise, supporting heavy-smokers to quit by various support mechanisms will lead to a utility gain of 0.0347. When applied at the population level, these small gains could translate into significant economic returns as explained below.

Our findings suggest that the more frequently one smokes, the worse quality of life they could expect from their smoking habit- regardless of other biological, clinical, lifestyle and socioeconomics. The absolute difference of 0.0347 in EQ-5D tariff between current heavy-smokers and ex-regular smoker is remarkable. Putting this into perspectives, there are currently 10 million smokers in England [56] of which according to our own data 27% (2.7 million) can be classified as heavy-smokers. Assuming that 6.4% of these smokers would receive a nicotine replacement therapy (NRT) prescription [57], 16% of whom will successfully quit at the end of the year [10], and conservatively assuming no deaths occurring in this group in this one year, this smoking cessation alone would save about 1000 QALYs at the end of the first year. If the NICE threshold for a QALY is used to value these benefits, NRT prescription alone could potentially save between £20-30 million (minus the costs of NRT provision) in one year.

Using the data provided in Table 3, a number of such policy simulations can be performed. Furthermore, these data can inform more robust economic evaluations of interventions that affect population smoking status (e.g. tobacco control) or are affected by it (e.g. lung cancer treatment). In particular, the information in the changes in utility due to smoking status in the population group that has no longstanding illness is valuable. By providing the utility values by age and gender, our estimates provides much more flexibility for cost-effectiveness researchers to model QALYs, compared to, for instance, the Scottish study [11] which provide a single estimate: the difference in ex-smokers and smokers (-0.0347) only. Our values are robust in the sense that they represent the net utility loss due to smoking. The standard deviation attached to each estimate will allow the modellers enough room to assess the uncertainty around their QALYs figures.

Finally, although the main driver of the paper is to provide utility values to inform the future economic evaluations, the findings around the EQ-5D domains warrant some interesting discussions. The fact that the frequency of severe conditions in our sample in all five domains was less than 4% mirrors the concern that EQ-5D is less able to pick up severe conditions [47]. Thus, combining 'some' and 'severe' problems into one category to model the effect that smoking status has on each of the five domains is not unreasonable. The findings that the degree of smoking, particularly more than 20 or more cigarettes a day, affects all domains is consistent with a priori expectations but what is new is the quantification of differential effect this has on the domains.

The fact that being a heavy-smoker is associated with 86% more likelihood of reporting some/severe problems in anxiety/depression compared with 42% in usual activity, coupled with the finding that quitting smoking (e.g. in the case of ex-regular smokers) does not affect self-care and usual activity but it continues to affect the other three domains, has two immediate implications for cessation interventions: (a) in order to improve quality of life among quitters, cessation services may need to combine other forms of support, e.g. facilitate access to mental health services; and (b) anxiety/depression and mobility are the two domains on which cessation services can have the greatest impact. Putting this into perspectives, encouraging heavy-smokers quit by various support mechanisms will lead to a massive 70% reduction in them reporting some/severe problems in anxiety/depression (49% in mobility). This is an important aspect to be communicated as an individual benefit of smokers in quitting campaigns. This is also supported by studies on the basic association of nicotine and anxiety/depression [58,59]. However, in order to assure sustained abstinence, it may be necessary to address/monitor mental health of those who attempt to quit right at the time of the intervention and beyond.

## Conclusion

Smoking is significantly and negatively associated with HRQoL in English general population. While the observed difference in EQ-5D mean score between individuals who never smoked and those who smoke at least 20 cigarettes a day is about 0.10, the actual difference after controlling for other biological, clinical, lifestyles and socioeconomic conditions is smaller (0.05) but is still significant. The implication is that supporting smokers quit will improve the population QALYs. The varying degree of the association between smoking profile and HRQoL need to be fed into the design of future economic evaluations where the intervention being evaluated affects (e.g. tobacco control) or is affected (e.g. treatment for lung cancer) by individuals' (or patients') smoking status. The net utility loss data due to various smoking profile reported in this paper is rich and can inform robust economic evaluations in the future.

## Competing interests

The authors declare that they have no competing interests.

## Authors' contributions

MV and SP designed the study, carried out the data analysis and constructed first draft of the manuscript with subsequent inputs and revisions from CMW and RL. All authors read and approved the final manuscript.

## Pre-publication history

The pre-publication history for this paper can be accessed here:

http://www.biomedcentral.com/1471-2458/12/203/prepub

## Supplementary Material

Additional file 1 **List of relevant studies exploring the association between HRQoL and smoking status **[11,45,46,49-55,60-63]. Click here for file

Additional file 2 **List of relevant studies exploring EQ-5D outcome of patients with smoking as confounding factor **[23,30,33,64-69]. Click here for file

Additional file 3 **Summary statistics of the sample**. Click here for file

Additional file 4 **OLS estimation of EQ-5D tariff**. Click here for file
